# Union Meeting of the New York Institute of Stomatology and the American Academy of Dental Science

**Published:** 1900-06

**Authors:** 

**Affiliations:** American Academy of Dental Science


### UNION MEETING OF THE NEW YORK INSTITUTE OF STOMATOLOGY AND THE AMERICAN ACADEMY OF DENTAL SCIENCE.

A union meeting of the New York Institute of Stomatology and the American Academy of Dental Science was held at Hotel Somerset, Boston, on the evening of March 21.

There were present sixteen members of the Institute and twenty-eight members of the Academy. The following gentlemen were also present as guests of the Academy: President C. W. Eliot, of Harvard University; Hon. J. J. Myers, Speaker of the Massachusetts House of Representatives; Rev. Dr. George A. Gordon, pastor of the Old South Church; Professor C. S. Minot, of the Harvard Medical School; and Harold Williams, M.D., Dean of Tufts College Medical and Dental Schools.

An hour of social intercourse was spent in the parlors of the hotel, after which a banquet was served, and an address of welcome delivered by Vice-President George F. Eames, of the Academy.

President Eliot was then introduced, and delivered a short address upon "The Newness of Dentistry."

(For President Eliot's address, see page 389.)

"Dentistry in its Relation to Medicine" was the subject assigned upon the programme to Dr. George S. Allan, of New York.

(For Dr. Allan's address, see page 406.)

Professor Minot took for his theme "The Union of Dental and Medical School."

(For Professor Minot's address, see page 393.)

The Rev. Dr. Gordon addressed the two societies upon "Dental Service as a Ministry to Life."

(For Dr. Gordon's address, see page 402.)

Dr. E. A. Bogue, of New York, read an interesting paper entitled "The Hand the Servant of the Brain."

(For Dr. Bogue's paper, see page 397.)

Dean Williams, in the course of his remarks, urged the necessity of a four-year course in the study of dental medicine and the establishment of a larger number of dental infirmaries for the benefit of the poorer classes.

Hon. J. J. Myers spoke in a happy vein upon "The Commonwealth as Benefactress and Beneficiary."

Dr. J. Morgan Howe, of New York, took for his text "Professional Dignity," and Dr. S. E. Davenport, of New York, gave utterance to the feeling entertained by all the members of the Institute present that the meeting had been a most pleasant and profitable one, expressing in conclusion the hope that a year hence the members of both societies might come together again in a similar union meeting in New York City.

On motion, adjourned.

Charles H. Taft,
*Editor American Academy of Dental Science.*

